# Parametric Optimization and Influence of Near-Dry WEDM Variables on Nitinol Shape Memory Alloy

**DOI:** 10.3390/mi13071026

**Published:** 2022-06-28

**Authors:** Rakesh Chaudhari, Aniket Kevalramani, Jay Vora, Sakshum Khanna, Vivek K. Patel, Danil Yurievich Pimenov, Khaled Giasin

**Affiliations:** 1 Department of Mechanical Engineering, School of Technology, Pandit Deendayal Energy University, Raysan, Gandhinagar 382007, India; rakesh.chaudhari@sot.pdpu.ac.in (R.C.); aniket.kmc18@sot.pdpu.ac.in (A.K.); vivekp@sot.pdpu.ac.in (V.K.P.); 2 Journal of Visualized Experiments, Delhi 110016, India; sakshum.khanna@gmail.com; 3 Department of Automated Mechanical Engineering, South Ural State University, Lenin Prosp. 76, 454080 Chelyabinsk, Russia; danil_u@rambler.ru; 4 School of Mechanical and Design Engineering, University of Portsmouth, Portsmouth PO1 3DJ, UK

**Keywords:** shape memory alloys, nitinol, optimization, near-dry wire electric discharge machining (WEDM), teaching–learning based optimization (TLBO) algorithm

## Abstract

Nitinol-shape memory alloys (SMAs) are widely preferred for applications of automobile, biomedical, aerospace, robotics, and other industrial area. Therefore, precise machining of Nitinol SMA plays a vital role in achieving better surface roughness, higher productivity and geometrical accuracy for the manufacturing of devices. Wire electric discharge machining (WEDM) has proven to be an appropriate technique for machining nitinol shape memory alloy (SMA). The present study investigated the influence of near-dry WEDM technique to reduce the environmental impact from wet WEDM. A parametric optimization was carried out with the consideration of design variables of current, pulse-on-time (T_on_), and pulse-off-time (T_off_) and their effect were studied on output characteristics of material removal rate (MRR), and surface roughness (SR) for near-dry WEDM of nitinol SMA. ANOVA was carried out for MRR, and SR using statistical analysis to investigate the impact of design variables on response measures. ANOVA results depicted the significance of the developed quadratic model for both MRR and SR. Current, and T_on_ were found to be major contributors on the response value of MRR, and SR, respectively. A teaching–learning-based optimization (TLBO) algorithm was employed to find the optimal combination of process parameters. Single-response optimization has yielded a maximum MRR of 1.114 mm^3^/s at T_on_ of 95 µs, T_off_ of 9 µs, current of 6 A. Least SR was obtained at T_on_ of 35 µs, T_off_ of 27 µs, current of 2 A with a predicted value of 2.81 µm. Near-dry WEDM process yielded an 8.94% reduction in MRR in comparison with wet-WEDM, while the performance of SR has been substantially improved by 41.56%. As per the obtained results from SEM micrographs, low viscosity, reduced thermal energy at IEG, and improved flushing of eroded material for air-mist mixture during NDWEDM has provided better surface morphology over the wet-WEDM process in terms of reduction in surface defects and better surface quality of nitinol SMA. Thus, for obtaining the better surface quality with reduced surface defects, near-dry WEDM process is largely suitable.

## 1. Introduction

The Shape memory alloys (SMA) are shape memory materials that can withstand immense deformations and yet return to their original shape by applied heat, stress or magnetic field [1,2]. This type of effect of regaining original shape is known as the shape memory effect (SME). SMAs exhibit superior thermomechanical properties [3,4,5]. Superelasticity of SMAs represents the property of regaining the original shape of material with the removal of applied external force. SMAs are widely preferred for engineering fields such as automobile, biomedical, aerospace, robotics [6,7]. Important applications for the industrial sector are fasteners and couplings generally for the military sector, cellular antennas, etc. [8]. The most commonly used SMAs are NiTi, CuZnAl, CoAl, NiMnGa, CuSn, FeMnSi, ZrCu, and CuZnNi. However, the instability and poor thermo-mechanic performance of these copper and iron-based SMAs have restricted their applications in certain areas [1]. Among others, nickel-titanium alloy also considered Nitinol was already employed in various engineering and industrial fields due to its enhanced characteristics such as high corrosion and wear resistance, biocompatibility, Superelasticity, SME, high strength, etc. [9,10,11]. Nitinol is also employed in multiple applications of biomedical fields, automotive sector, sensors, MEMS devices, actuators, structural elements, oil industries, robotics, aerospace components, etc. [12,13]. Therefore, precise machining of Nitinol SMA plays a vital role in achieving better surface roughness, higher productivity and geometrical accuracy for the manufacturing of devices [14,15]. The machinability of Nitinol with conventional methods was observed to be a non-effective technique due to the formation burrs at the machined surface, poor surface roughness, and high tool wear [16,17]. One of the prime reasons behind this was the higher strength and hardening of the nitinol SMA [18,19]. Another reason which possesses the difficulties includes high chemically active material which in turn results in tool failure, and low thermal conductivity [20,21]. 

To overcome difficulties faced during machining of SMAs using traditional techniques, non-conventional machining techniques are considered good alternatives since the workpiece and tool are not in contact with each other. Among other methods, Wire electric discharge machining (WEDM) has proven to be an appropriate technique for machining nitinol SMAs [22,23]. The WEDM technique works based on spark generation and erosion between electrode and workpiece [24]. In WEDM process, the material is melted and vaporized by repeated electrical discharges in presence of a suitable dielectric medium [25,26]. It uses wire as an electrode and the dielectric fluid which firstly acts as an insulator and later gets ionized by increasing the amount of voltage [27]. This further increases the electrical discharges (sparks) which in turn helps in increasing the material removal rate (MRR). Numerical control of the wire electrode has made WEDM process to be vastly suitable for creating the complex shape profiles of the workpiece [28,29]. Kulkarni et al. [30] employed WEDM process to study surface integrity aspects for Nitinol SMA. They utilized RSM models to generating the relationship between design variables and responses. Higher erosion with good surface morphology was obtained at optimal parameter settings. Thus, the monitoring and controlling of the machining process should be carried out properly for better machining efficiency, and to prevent wire breakage and surface quality. WEDM can provide a good surface finish, and good machining efficiency for machining complex shapes [31,32]. However, WEDM operation utilizes dielectric fluid which is a key factor in environmental issues. To overcome this issue, near-dry machining process can be an efficient and effective way by means of providing negligible health hazards [33]. Near dry machining replaces the EDM oil with a mixture of compressed air and water [34]. In addition to the environmental issues, machining characteristics such as productivity and surface quality should not be compromised. Near dry WEDM (NDWEDM) process was found to be capable of providing enhanced machining results for machining of hard materials [35,36]. NDWEDM process makes use mixture of minimal dielectric fluid and gas/air. Minimal use of deionized water along with larger proportion of compressed air/gas was found to be effective to enhance the NDWEDM performance under eco-friendly atmosphere [37]. Researchers have reported several studies to examine the implications of NDWEDM technique on machining. A suitable amalgamation of design variables for WEDM can be achieved by finding an optimal solution to opposing responses [38,39]. To find an optimal solution for contradictory responses, new optimization techniques were invented wherein amendment of algorithm-specific parameters is not required [40]. Teaching–learning-based optimization (TLBO) algorithm is one such technique which does not require fine-tuning of variables [41,42] and is found to be easy to execute [43]. Researchers have successfully used this techniques in various fields along with problems related to manufacturing sectors [44,45]. 

Kumar et al. [33] studied the effect of NDWEDM on nickel-based superalloy-Monel. They utilized a blend of compressed air and deionized water at a suitable proportion for obtaining the near-dry condition. Effect of pulse-on-time (T_on_), voltage, pulse-off-time (T_off_), and wire feed has been studied for responses of material removal rate (MRR), and surface roughness (SR). Lower values of T_on_ are key influencing factors for desired better surface finish. Comparison of NDWEDM with wet WEDM results yielded substantial improvement in SR for NDWEDM process. Liu et al. [46] concluded that the machined trim cut samples of Nitinol using WEDM machining have lower SR and minimal white layer as compared to main cut Nitinol samples. Dhakar et al. [47] studied near dry EDM and wet-EDM with inputs of T_off_, current, T_on_ to evaluate the MRR of high-speed steel (HSS). A correlation between design variables and output parameters for NDEDM and wet EDM has been developed. The current was established as the largest dominating variable for enhancing the MRR during near-dry EDM and wet EDM processes. Wet EDM process was found to produce more gas emission concentration in comparison with NDEDM. For achieving the required desirable machining performance, Kao et al. [48] have performed NDWEDM in which a liquid and gas mixture was used as a dielectric fluid and also has the advantage to modify properties of the dielectric medium and liquid concentration. WEDM and EDM drilling were examined under all three variants of the EDM process such as dry, wet, and near-dry EDM. Their obtained results have depicted higher MRR for near dry WEDM process. Yu et al. [49] compared the dry-EDM machining performance of cemented carbide with wet EDM technique. Their obtained results have shown an improvement in machining efficiency and drop-in tool wear rate by implementing dry EDM process. For obtaining the good machining efficiency at minimal discharge energy and simultaneously better surface quality with low environmental problems, Boopathi et al. [50] used near dry EDM for conducting experiments on HSS-M2 with a mixture of liquid with air and liquid with oxygen as a dielectric medium. The effect of design variables has been studied on MRR and SR by employing Taguchi method. Their obtained results have shown that the use of a moderate proportion of air-mist pressure increases MRR with subsequently reduces SR. Gholipoor et al. [51] have compared output characteristics of MRR, TWR, and SR obtained by near-dry EDM with wet EDM and dry EDM for machining of SPK steel. Scanning electron microscopy (SEM) was used to analyse the surface integrity of this process and compare it to wet and dry EDM processes. SEM micrographs demonstrated that the surface morphology of obtained surface by NDEDM was better in comparison with the surfaces obtained at dry and wet EDM process as the surface has largely reduced micro-cracks and craters with the use of NDEDM technique. Boopathi and Sivakumar [52] optimized the performance of near-dry WEDM process of HSS by using a multi-objective evolutionary algorithm. They have utilized air-mist dielectric condition to study the influence of design variables such as T_on_, gap voltage, current, T_off_, and current on MRR and SR. ANOVA results has shown that current was having highest impact on deciding the values of MRR and SR. Moderate air-mist pressure was found to have substantial effect for increase in MRR and simultaneous improvement in surface quality. Regression equations were developed to find correlation between design variables and responses. They employed Pareto fronts to solve the contradictory situation among responses of MRR, and SR. 

Till now, most of the research has been performed on studying the effect of near-dry WEDM variables and their impact on machining characteristics for steels and other alloys. However, to the best of the authors’ knowledge, experimental investigations, and multi-objective optimization of near-dry WEDM process for nitinol SMA not yet conveyed. The current study investigated the performance of near-dry WEDM process with consideration of WEDM parameters of T_on_, T_off_, and current for Nitinol SMA. Box–Behnken design was utilized to conduct the experiments and mathematical correlations were developed between output characteristics (MRR and SR) and design variables. ANOVA was carried out for MRR, and SR using statistical analysis to investigate the impact of design variables on response measures. ANOVA results depicted the significance of the developed quadratic model for both MRR and SR. Current, and T_on_ were found to be major contributors on the response value of MRR, and SR, respectively. TLBO algorithm has been executed for single-objective and multi-objective optimization of MRR, and SR. Near-dry WEDM process yielded an 8.94% reduction in MRR in comparison with wet-WEDM, while the performance of SR has been substantially improved by 41.56%. Lastly, scanning electron microscopy was utilized to study the surface morphology of obtained surfaces from near-dry WEDM and wet WEDM. Thus, for obtaining the better surface quality with reduced surface defects, near-dry WEDM process is largely suitable. Authors believes that current study will be useful for machining of nitinol SMA for acquiring good surface quality. 

## 2. Materials and Methods

**Figure 1:**
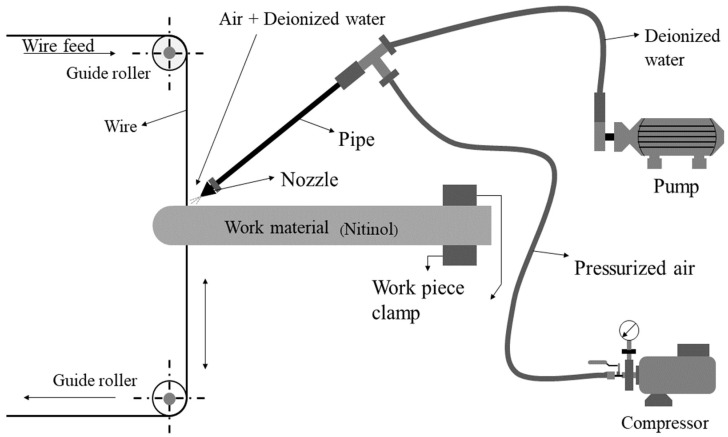
Schematic representation of near-dry WEDM setup.

Concord WEDM DK 7732 machine was employed in the present work to conduct the experiments by using near-dry WEDM process which is an advanced variant of WEDM technique. Nitinol rod with 10 mm diameter were considered as work material in the present study. The selected work material of nitinol contains 55.8% of nickel and reminder is titanium. Molybdenum wire having a diameter of 0.18 mm was selected as a tool material. With respect to the use of dielectric medium, WEDM process consists of three main process namely, wet-WEDM, dry WEDM, and near dry WEDM. In case wet WEDM process, only dielectric has been used, while dry WEDM makes use of only compresses gas as dielectric medium. Near dry WEDM process consist of both these medium, i.e., minimum quantity of dielectric fluid and compressed gas. In current study, a mixture of dielectric fluid (minimum quantity of liquid) and compressed air was used as an air-mist dielectric medium. Figure 1 shows a schematic diagram of near-dry WEDM experimental setup. 

Based on the machine limits, preliminary trials, and recent literature conducted on machining of near-dry WEDM and nitinol SMA, and preliminary experimentations, T_on_, T_off_ and Current were selected as design variables for studying their effects on MRR, and SR. The three levels of design variables for T_on_ includes 35, 65, and 95 µs; T_off_ includes 9, 18, and 27 µs; Current includes 2, 4, and 6 A. The experimental matrix was formed by using the Box–Behnken design (BBD) technique. By following the BBD matrix, 15 trails were completed with the variation in design variables at three levels. BBD design of RSM was used to obtain an optimum response by using a series of designed experiments. Another purpose of implementing BBD design was to develop mathematical correlations between input and output parameters [53]. RSM was employed for the reduction in the experimental trials which avoids additional time and cost required for material [54,55]. To study the statistical analysis of design variables for responses of MRR, and SR, Minitab 17 was employed. 

By following the BBD design matrix, experimental trials were performed thrice by taking average value of repetitions. As per Equation 1, the material erosion rate was evaluated in mm^3^/s.

(1) $$\mathrm{MRR}\;=\frac{\Delta W*1000}{\rho *t}$$

where  ΔW represents eroded material in gram, t depicts the time in second, and ρ represents the density of the nitinol SMA (6.5 g/cm^3^). 

**Figure 2:**
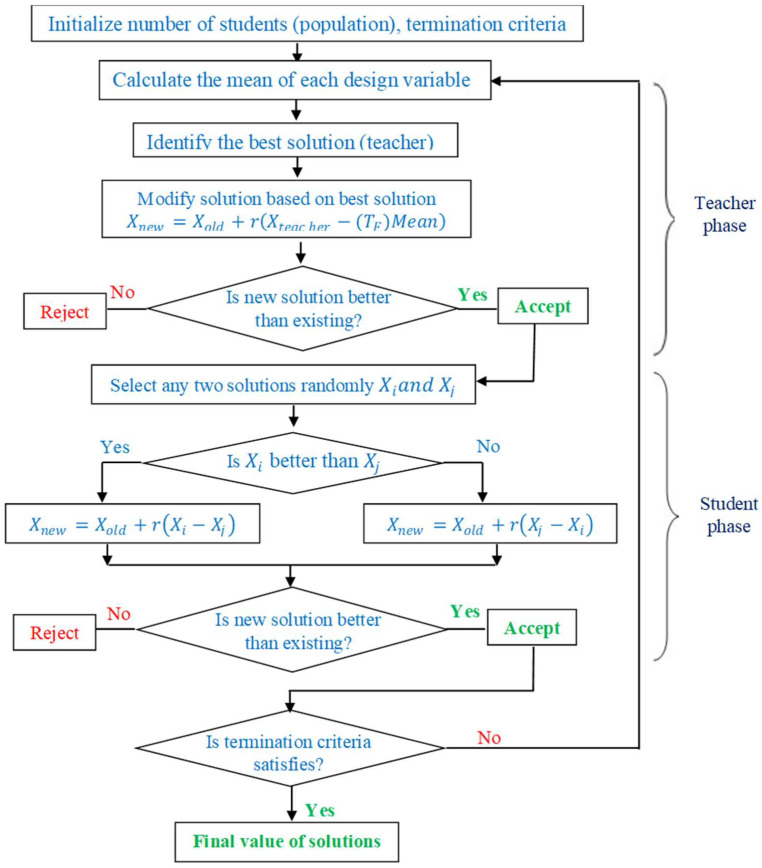
TLBO algorithm [57].

SR was determined by employing Mitutoyo make Surftest SJ-410 with the consideration of 0.8 mm as cut-off length. Measurement of SR was performed thrice at various locations by taking average value of repetitions. SEM was employed to investigate the effect of near-dry WEDM and wet WEDM processes on surface morphology. TLBO algorithm developed by Vivek and Vimal [56] was employed to find the optimal combination of process parameters. TLBO operates on the principle of teaching and learning activities of students in a group. During the execution of the algorithm, the teacher tries to achieve the performance of class students adjacent to the student securing the highest grade by means of shifting the means of topper student grade. During the teacher phase, teacher guides the students of class. Learner phase consists of an interaction of students among themselves. Working principle of TLBO technique was depicted in Figure 2. 

## 3. Results and Discussions

Table 1 depicts the experimental matrix as per the selected Box–Behnken design, design variables with their levels and evaluated readings of MRR, and SR. A mathematical relationship between design variables and responses has been developed by using RSM approach and by employing Minitab v17 (Bangalore, India). ANOVA was then carried out by using Minitab v17 for statistical analysis and to investigate the impact of design variables on response measures. Further, main effect plots were used to understand the influence of design variables on deciding the values of MRR, and SR. These main effect plots highlight the suitable levels of design variable for a specifically required output. 

### 3.1. Generation of Non-Linear Regression Equations for Responses

A mathematical correlation has been developed between design variables and response measured with the help of RSM approach. Regression equations were generated by using Minitab v17 software. Equations (2) and (3) depicts the generated regression equations for MRR, and SR, respectively, by employing the stepwise approach which eliminates the non-significant terms from the model as they do not have any meaningful impact on response values.

(2) $$\begin{array}{ll} \mathrm{MRR}=1.0813 & +0.00079\cdot T_{\mathrm{on}\;}-0.02512\cdot T_{\mathrm{off}}-0.1338\cdot \mathrm{Current}\\ & +0.000040\cdot T_{\mathrm{on}}\cdot T_{\mathrm{on}\;}+0.000265\cdot {\;T}_{\mathrm{off}}\cdot T_{\mathrm{off}\;}+0.01456\\ & \cdot \;\mathrm{Current}\cdot \mathrm{Current}-0.000207\cdot {\;T}_{\mathrm{on}}\cdot \mathrm{Current}+0.002777\\ & \cdot {\;T}_{\mathrm{off}}\cdot \mathrm{Current} \end{array}$$

(3) $$\begin{array}{ll} \mathrm{SR}=5.379-0.04107 & \cdot T_{\mathrm{on}\;}-0.0944\cdot T_{\mathrm{off}}-0.046\cdot \mathrm{Current}+0.000238\cdot T_{\mathrm{on}}\\ & \cdot T_{\mathrm{on}\;}+0.0517\cdot \;\mathrm{Current}\cdot \mathrm{Current}+0.001065\cdot {\;T}_{\mathrm{on}}\cdot {\;T}_{\mathrm{off}} \end{array}$$

### 3.2. ANOVA for MRR and SR

ANOVA was carried out for MRR, and SR by using Minitab v17 for statistical analysis and to investigate the impact of design variables on response measures. A confidence level of 5% was employed to investigate the effect of design variables [58]. To have a significance of an input variable on the output variable, it is desired to have the P-value be less than 0.05 [59,60]. Table 2 shows the ANOVA for the response measure of MRR. A stepwise approach with α value equivalent to 0.15 was developed which eliminates the non-significant terms having from the model as they do not have any meaningful impact on response values. ANOVA results of Table 2 describe the statistical significance of the quadratic model of MRR as the regression model term, linear model, square interaction, and 2-way interactions are all significant. In addition to this, the non-significance of lack of fit with a P-value of 0.257 signified the robustness and adequacy of the developed model for MRR [57]. According to P-values, statistically significant factors include all the linear terms such as T_on_, T_off_, current; all square terms T_on_ × T_on_, T_off_ × T_off_, current × Current; interaction term T_off_ × Current. A major contributor to deciding the response value of MRR was found to be T_on_ followed by T_off_, and current. R^2^ value adjacent to one is considered as acceptability of regressions to predict the response value. The obtained values of R^2^ with 0.99447 and Adj. R^2^ with 0.9876 depicts the adequacy and fitness of the model. The standard deviation of 0.0147 has been observed for MRR response. It reveals that theoretical maximum deviation for MRR will be only 0.0147 from the mean value of MRR.

Statistical analysis from ANOVA for SR was shown in Table 3. Non-significant terms from the model have been eliminated by following the stepwise approach with an α value equivalent to 0.15 as these eliminated terms do not have any meaningful impact on response values. ANOVA results of Table 3 describe the statistical significance of the quadratic model of SR as the regression model term, linear model, square interaction, and 2-way interactions are all significant. In addition to this, the non-significance of lack of fit with a P-value of 0.193 signified the robustness and adequacy of the developed model for MRR [61]. According to P-values, statistically significant factors include all the linear terms T_on_, T_off_, current; square terms T_on_ × T_on_, current × current; interaction term T_on_ × T_off_. A major contributor to deciding the response value of MRR was found to be currently followed by T_on_ and then T_off_. R^2^ value close to unity is considered as acceptability of regressions to predict the response value. The obtained values of R^2^ (0.9855) and Adj. R^2^ (0.9746) closed to unity has depicted the adequacy and fitness of the model. The standard deviation of 0.1048 has been observed for SR response. It reveals that theoretical maximum deviation for MRR will be only 0.1048 from the mean value of SR. These obtained results from ANOVA for both the responses of MRR and SR have suggested the suitability of the developed model for the prediction of upcoming response measures. However, it is mandatory to validate the results obtained from ANOVA by generating the residual plots. 

### 3.3. Residual Plots for MRR and SR

**Figure 3:**
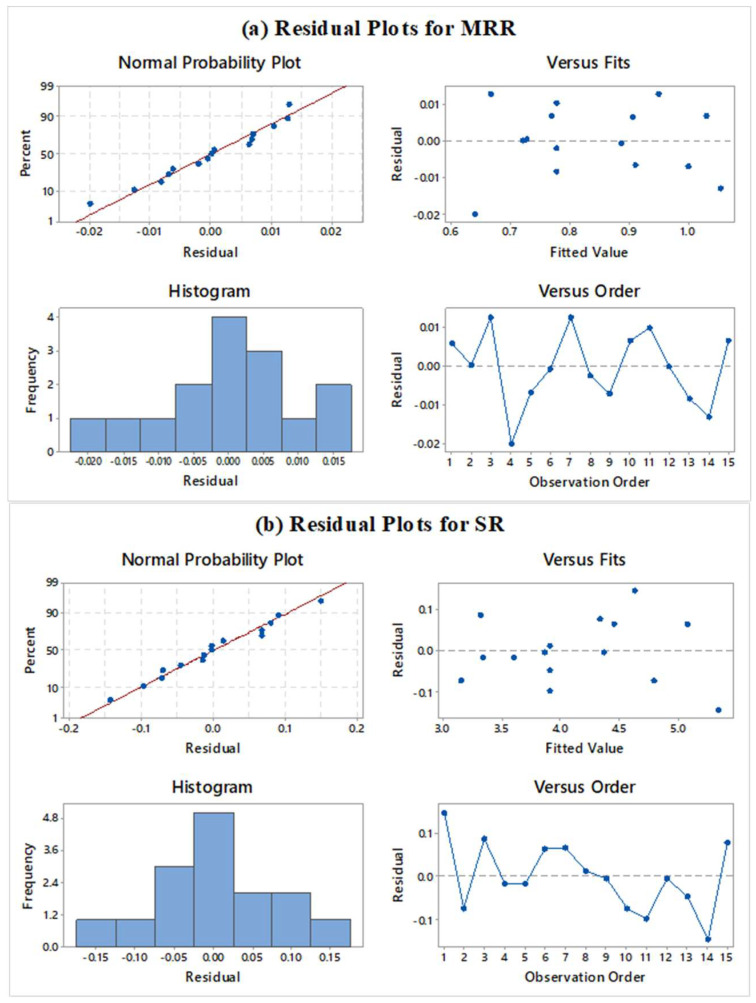
Residual plots for (**a**) MRR, and (**b**) SR.

Figure 3a,b depict the residual plots for response variables. Successful verification of residual plots produces the successful outcomes from ANOVA results [62,63]. Residual plots consist of four plots such as normal probability, versus fits, histogram, and versus order plot. From Figure 3a,b, the normal probability shows the plot between the percentages versus the residual. Normality plot verifies that entire the residuals are on a straight line. This shows that the model assumptions are correct, and the errors are normally distributed [64]. Randomized residuals were observed in the versus plot which suggests the suitability of the test [65]. Figure 3a,b validate the results of versus fits plot for both the responses. The histogram has shown a parabolic curve which depicted verification of ANOVA results [66]. In the last plot of residual versus observation orders, the absence of any pattern fulfils the key requirement of significant ANOVA [67]. Figure 3a,b do not depict any kind of formation of pattern for all responses which suggest good ANOVA results. Therefore, ANOVA test results can now be treated as effective and fit for developed regression models as residual plots has fulfilled the assumptions.

### 3.4. Effect of WEDM Variables on Responses

Main effect plots were derived by using Minitab v17 to investigate the impact of WEDM parameters on MRR, and SR. Desired output performance (maximum/minimum) of the responses in the selected levels can be efficiently represented by these main effect plots. By considering the requirement of higher productivity, the objective for MRR response was considered as maximization. Lower SR is anticipated for a better surface quality. So, minimization criteria were assigned to the SR response. The *X*-axis depicts the individual variable while *Y*-axis represents the output responses of MRR and SR. 

**Figure 4:**
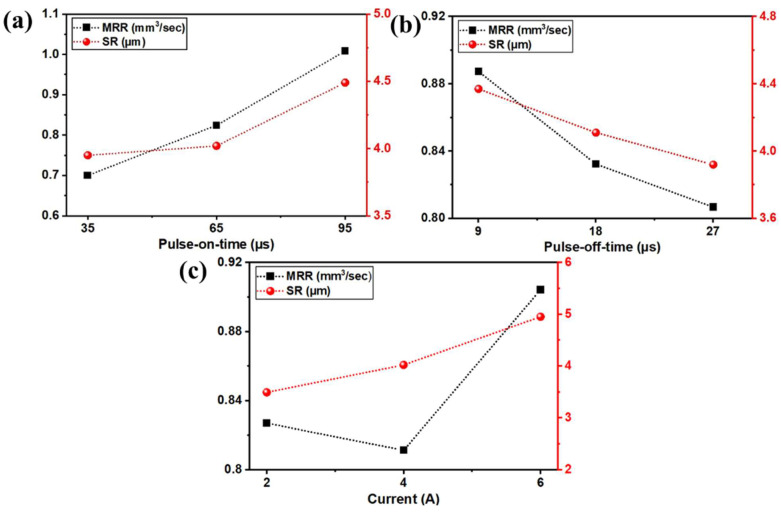
Impact of WEDM variables on MRR, and SR for (**a**) Pulse-on-time, (**b**) Pulse-off-time, and (**c**) Current.

Figure 4a shows the influence of the T_on_ on MRR and SR. Both the selected responses MRR, and SR were observed to be increased with the rise in T_on_ from 35 µs to 95 µs. As per ANOVA, T_on_ was having most dominating factor in affecting the MRR response. MRR was increased from 0.7002 mm^3^/s to 1.0082 mm^3^/s with a subsequent rise in T_on_. The reason for the increase in MRR is the efficient flushing at the interelectrode gap (IEG) owing to the substantial flushing pressure during NEWEDM process [68]. This efficient flushing further enhanced spark formations which in turn increased the MRR [69]. Recurring spark formation leads to the melting and vaporization of work material thereby, erosion rate during machining [70]. Increased T_on_ subsequently enriches thermal energy which in turn enhances the sparking frequency [71,72]. This is the additional factor for obtaining higher MRR. An extensive conducted by Boopathi [34] has concluded similar observations for increased MRR with rise in T_on_ value. SR was increased with rise in T_on_ value. Production of higher frequency sparks and thermal energy due to the escalation in the value of T_on_ has generated larger and deeper craters on the work surface [73,74]. Kumar et al. [33] has found a similar trend of increased SR with rise in T_on_. The formations of larger craters diminish the surface quality and thus, SR also increased during the NDWEDM process [75]. This showed the different levels of T_on_ for acquiring the higher MRR and lower SR. For obtaining higher MRR and lower SR, desired levels of T_on_ were established as 95 µs, and 35 µs, respectively. 

The impact of the design variable T_off_ on MRR and SR can be observed in Figure 4b. The declining trend can be seen for both responses of MRR, and SR with an increasing value of T_off_. Increasing pulse duration T_off_ from 9 µs to 27 µs has reduced MRR from 0.8873 mm^3^/s to 0.8067 mm^3^/s and improved SR from 4.37 µm to 3.92 µm. The reason for such declined value of MRR was due to the reduction in sparking frequency. T_off_ depicts the interval between the occurrences of two successive sparks [76]. Thus, an increase in T_off_ will have a negative effect on sparking between IEG. Reduction in sparking subsequently reduces the melting and vaporization of work material and thus, the erosion rate diminishes by leading to lower MRR [77]. Results obtained in present work are in line with the conclusion drawn by Manjaiah et al. [8] for drop in MRR. On the other hand, a rise in T_off_ has a positive effect on the SR of work material. Declined sparking in IEG also drops the temperature owing to a rise in T_off_. This will further reduce the thermal and discharge energy and will create smaller craters [78]. Due to this reason, the quality of the work surface has been improved and a smooth surface was obtained by observing a drop in SR value [79]. Fuse et al. [80] has shown a similar trend of drop in SR value with increase in T_off_. This showed the different levels of T_off_ for acquiring the higher MRR and lower SR. The desired levels of T_off_ were established as 9 µs, and 27 µs for MRR, and SR, respectively. 

Figure 4c represented the influence of the current on MRR and SR. Both the selected responses MRR, and SR were observed to be increased with the rise in current from 2 A to 6 A. As per ANOVA, the current was the most influential factor in the SR response. Increasing current from 2 A to 6 A has improved MRR from 0.8113 mm^3^/s to 0.9041 mm^3^/s and decreased the quality of the surface by increasing SR from 3.49 µm to 4.95 µm. The reason behind the improvement in MRR values is discharge energy. Enhancement in current further improved the discharge energy. It is further converted into thermal energy which enhances the sparking frequency during NDWEDM [81]. The formation of recurring spark leads to the melting and vaporization of work material thereby, erosion rate during machining [82]. Thus, MRR was improved with a rise in current. Similar conclusion was drawn in the study carried out by Dhakar et al. [47]. For SR response, the current was found to be the highest contributing factor. A negative effect of a rise in the current on SR can be seen in Figure 4c. As escalation in current gives rise to thermal energy, bigger and deeper craters get formed on work material [83]. Thus, a drop in SR with rising in current was depicted due to the formation of tiny craters. This main effect plot has shown the different levels of current for acquiring the higher MRR and lower SR. For obtaining higher MRR and lower SR, desired levels of current were established as 6 A, and 2 A, respectively. 

### 3.5. Optimization Using TLBO Technique

TLBO algorithm has been executed for single-objective and multi-objective optimization of MRR, and SR. TLBO is one such technique which does not require fine-tuning of variables, and found to be easy to execute. Results of main effect plots have depicted extreme opposite levels of design variables to attain anticipated levels of responses. In the present study, the objective for MRR response was considered as maximization by considering the requirement of higher productivity. On the other hand, minimization criteria were assigned to SR response as lower SR is always desirable to acquire a better quality of the machined components. TLBO algorithm is fast and easy to implement. During the execution of the algorithm, MRR, and SR were considered positive entities. Levels of design variables employed during execution of TLBO include T_on_: 35 µs ≤ T_on_ ≥ 95 µs; T_off_: 9 µs ≤ T_off_ ≥ 27 µs; Current: 2 A ≤ current ≥ 6 A. 

Results of single-response optimization have been represented in Table 4. Single-response optimization has yielded a maximum MRR of 1.114 mm^3^/s at T_on_ of 95 µs, T_off_ of 9 µs, current of 6 A. Least SR was obtained at T_on_ of 35 µs, T_off_ of 27 µs, current of 2 A with the predicted value of 2.81 µm. Validation trials of these optimized results were carried out by performing the experiments at the obtained design variables. Predicted and actual determined values from trials were represented in Table 4. It can be observed that all the experimentally obtained response measures were in line with the predicted results showing a minimum error within the acceptable range. This has shown acceptability of proposed regression models with TLBO for the near-dry WEDM process. However, single-response optimal results have shown extreme opposite levels of design variables for attaining maximum MRR, and minimum SR. The suitable amalgamation of design variables for WEDM can be achieved by finding an optimal solution to opposing responses. To find the optimal solution for contradictory responses, a set of non-dominated optimum solutions provided by Pareto fronts has proven to be very effective. From Pareto fronts, user can select any optimal value as per their requirement with near-dry WEDM. 

**Figure 5:**
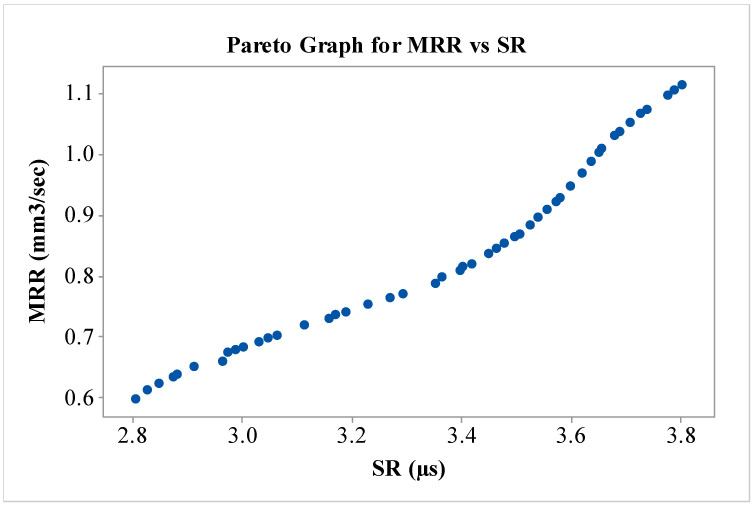
Pareto graph for MRR vs. SR.

Multi-response TLBO (MOTLBO) algorithm has been utilized to produce the simultaneous optimal levels of MRR, and SR. Fifty Pareto optimal points were generated and each Pareto point depicts the distinctive optimal result. Table 5 represents the generated solutions from the MOTLBO algorithm along with the values of design variables. Pareto curve has also been generated to understand the behavior of variation of MRR, and SR response measures. Figure 5 denotes the generated Pareto graph from unique and independent values of MRR, and SR. The nature of the Pareto curve depicts the conflicting nature between MRR and SR. Pursuant to this Pareto points will be useful selecting the corresponding levels of NDWEDM variables. By picking five random points from Table 5, validation trials of these optimized results were carried out. For all the performed experiments, an acceptable error of less than 5% was noticed among predicted and experiment trials. Thus, the obtained results have established an acceptability of the developed regression models with TLBO technique for near-dry WEDM process.

### 3.6. Comparison Study Near-Dry WEDM with Wet WEDM Process

To investigate the performance of the NDWEDM process with wet WEDM, a case study has been considered with the objective function as represented in the equation. For assigning the identical significance to both the responses MRR, and SR, a multi-response optimization methodology was utilized with an equal weight of 0.5 to output responses by using the TLBO algorithm.

(4) $$\mathrm{Obj}\;=w_{1}\cdot \left(\mathrm{MRR}\right)+w_{2}\cdot \left(\mathrm{SR}\right)$$

The objective function has yielded optimized values of MRR, and SR as 0.815 mm^3^/s, and 3.41 µm, respectively, at design variables of T_on_ of 71 µs, T_off_ of 20 µs, current of 2 A. validation trial was again conducted for the verification of these results and it has shown the actual MRR, and SR of 0.829 mm^3^/s, and 3.29 µm, respectively. Now, for the comparison of these obtained results from the NDWEDM process, another experiment was carried out at the same set of parameters by using the wet-WEDM process. During the wet-WEDM process supply of air has been removed and only deionized water was used as a medium. Experimental results obtained from the wet-WEDM process have produced MRR, and SR of 0.761 mm^3^/s, and 5.63 µm, respectively. A small reduction in MRR with a decrease of 8.94% has been observed for the NDWEDM process in comparison with wet-WEDM. Higher MRR for the wet-WEDM process was due to the fact that dielectric fluid is having higher thermal conductivity as compared to the air-mist mixture [51]. Lower thermal conductive materials are having less impact on melting and vaporization during the machining process [33]. This in turn reduces the rate of erosion and thus, MRR. Another reason for higher MRR in the case of wet-WEDM is that it has improved sparking frequency as compared to near-dry WEDM. The reason for this is that NDWEDM provides dielectric fluid in the form of small droplets [48]. However, the performance of SR has been substantially improved by 41.56% with the use of the NDWEDM process. This is due to the fact that the lower viscosity of the NDWEDM process reduces the current density [33]. This in turn results in the formation of tiny shallow craters and produces better surface quality [84]. Another reason for lower SR during NDWEDM was due to improved flushing of debris particles from IEG [85,86]. 

### 3.7. Surface Morphology of Near-Dry WEDM and Wet WEDM Process

**Figure 6:**
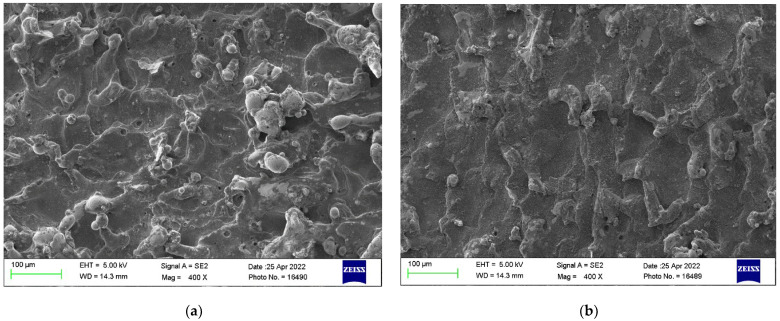
SEM micrograph at T_on_ of 71 µs, T_off_ of 20 µs, current of 2 A for (**a**) wet-WEDM, and (**b**) near-dry WEDM.

The surface morphology of the machined surface plays an important role to understand the significance of design variables and the machining process. Machined surfaces obtained at design variables of T_on_ of 71 µs, T_off_ of 20 µs and current of 2 A were selected to study the surface morphology of both the processes of NDWEDM and wet-WEDM. Figure 6a,b depict the SEM images for the machined surface obtained by using the wet-WEDM and NDWEDM processes, respectively. Figure 6a shows the large presence of surface defects such as globules and deposition of solidified material, micro-voids, and micro-cracks. This was due to the high thermal energy generated during the wet-WEDM process [34]. This high thermal energy generated enhances the intensity of the spark and thus, it produces a higher temperature at IEG. This in turn evaporates more material and generates high surface deviations in the form of micro-voids, deposition of solidified material, and micro-cracks [87,88]. On the other hand, the machined surface produced by using the NDWEDM process, as per Figure 6b, depicts lower surface deviations. This is due to the fact that the lower viscosity of the NDWEDM process reduces the current density [89]. This in turn results in the formation of tiny shallow craters and produces better surface quality by reducing the surface defects such as micro-voids, deposition of solidified material, and micro-cracks [84]. Another reason behind this was the improved flushing of debris particles from IEG [51]. Therefore, low viscosity, reduced thermal energy at IEG, and improved flushing of eroded material for air-mist mixture during NDWEDM have provided better surface morphology over the wet-WEDM process in terms of reduction in surface defects and better surface quality of nitinol SMA.

## 4. Conclusions

In current study, near-dry machining process was used to overcome the environmental issues by means of providing negligible health hazards. Parametric optimization was carried out by employing the TLBO algorithm. The influence of near-dry WEDM technique was studied to relieve environmental issues related to wet WEDM with the consideration of T_on_, T_off_, and current as design variables. Following significant conclusions can be drawn from the present study.

- The mathematical non-linear regression equations obtained from experimental results were found to be effective for prediction of responses.
- ANOVA results depicted the statistical significance of the quadratic model for both responses MRR, and SR as the regression model term, linear model, square interaction, and 2-way interactions are all significant. A major contributor to deciding the response value of MRR was found to be T_on_ followed by T_off_, and current, while for SR, the current was having a major contributing element followed by T_on_ and then T_off_.
- R^2^ values closed to unity signified the adequacy and fitness of the MRR, and SR model. Non-significance of lack of fit for both MRR and SR has again signified the robustness and adequacy of the developed model. All four residual plots for MRR and SR have verified the good statistical analysis for ANOVA and the outcome of developed regression equations.
- TLBO algorithm has been executed for single-objective and multi-objective optimization of MRR, and SR. Single-response optimization has yielded a maximum MRR of 1.114 mm^3^/s at T_on_ of 95 µs, T_off_ of 9 µs, current of 6 A. Least SR was obtained at T_on_ of 35 µs, T_off_ of 27 µs, current of 2 A with the predicted value of 2.81 µm.
- Pareto fronts presented a trade-off between two conflicting objectives, and manufacturers can select any point on the front.
- The objective function for near-dry WEDM has yielded optimized values of MRR, and SR as 0.815 mm^3^/s, and 3.41 µm, respectively, at design variables of T_on_ of 71 µs, T_off_ of 20 µs, current of 2 A. Experimental results obtained from the wet-WEDM process have produced MRR, and SR of 0.761 mm^3^/s, and 5.63 µm, respectively.
- Near-dry WEDM process yielded a small reduction in MRR with an 8.94% decrease in comparison with wet-WEDM. However, the performance of SR has been substantially improved by 41.56%.
- SEM micrographs were used to study the surface morphology of obtained surfaces from near-dry WEDM and wet WEDM. Low viscosity, reduced thermal energy at IEG, and improved flushing of eroded material for air-mist mixture during NDWEDM has provided better surface morphology over the wet-WEDM process in terms of reduction in surface defects and better surface quality of nitinol SMA.
- Thus, for obtaining the better surface quality with reduced surface defects, near-dry WEDM process is largely suitable. Authors believes that current study will be useful for machining of nitinol SMA for acquiring good surface quality.

## Figures and Tables

**Table 1:** The experimental matrix as per BBD and response measures of MRR, and SR.

Run Order	T_on_ (µs)	T_off_ (µs)	Current (A)	MRR (mm^3^/s)	SR (µm)
1	65	27	6	0.91295	4.77
2	65	27	2	0.73005	3.08
3	35	18	2	0.68045	3.41
4	35	27	4	0.62155	3.32
5	65	9	2	0.90365	3.59
6	65	9	6	0.88660	5.14
7	95	27	4	0.96255	4.52
8	65	18	4	0.77655	3.92
9	95	18	2	0.99355	3.86
10	35	18	6	0.77655	4.72
11	65	18	4	0.78895	3.81
12	35	9	4	0.72230	4.36
13	65	18	4	0.77035	3.86
14	95	18	6	1.04005	5.19
15	95	9	4	1.03695	4.41

**Table 2:** ANOVA for MRR.

Source	DF	SS	MS	F	P	Significance
**Model**	8	0.242278	0.030285	139.89	0.000	#
**Linear**	3	0.214690	0.071563	330.56	0.000	#
**T_on_**	1	0.189805	0.189805	876.73	0.000	#
**T_off_**	1	0.012993	0.012993	60.01	0.000	#
**Current**	1	0.011893	0.011893	54.93	0.000	#
**Square**	3	0.016978	0.005659	26.14	0.001	#
**T_on_ × T_on_**	1	0.004727	0.004727	21.83	0.003	#
**T_off_ × T_off_**	1	0.001698	0.001698	7.84	0.031	#
**Current × Current**	1	0.012530	0.012530	57.88	0.000	#
**2-Way Interaction**	2	0.010610	0.005305	24.50	0.001	#
**T_on_ × Current**	1	0.000615	0.000615	2.84	0.143	*
**T_off_ × Current**	1	0.009995	0.009995	46.17	0.000	#
**Error**	6	0.001299	0.000216			#
**Lack of fit**	4	0.001120	0.000280	3.12	0.257	*
**Pure error**	2	0.000179	0.000090			
**Total**	14	0.243577				

R^2^ = 99.47%; R^2^ (Adj.) = 98.76%; # = Significant term; * = Non-Significant term.

**Table 3:** ANOVA for SR.

Source	DF	SS	MS	F	P	Significance
**Model**	6	5.95782	0.99297	90.40	0.000	#
**Linear**	3	5.31992	1.77331	161.45	0.000	#
**T_on_**	1	0.58861	0.58861	53.59	0.000	#
**T_off_**	1	0.40951	0.40951	37.28	0.000	#
**Current**	1	4.32180	4.32180	393.48	0.000	#
**Square**	2	0.30727	0.15364	13.99	0.002	#
**T_on_ × T_on_**	1	0.17047	0.17047	15.52	0.004	#
**Current × Current**	1	0.15874	0.15874	14.45	0.005	#
**2-Way Interaction**	1	0.33063	0.33063	30.10	0.001	#
**T_on_ × T_off_**	1	0.33063	0.33063	30.10	0.001	#
**Error**	8	0.08787	0.01098			#
**Lack of fit**	6	0.08180	0.01363	4.49	0.193	*
**Pure error**	2	0.00607	0.00303			
**Total**	14	6.04569				

R^2^ = 98.55%; R^2^ (Adj.) = 97.46%; # = Significant term; * = Non-Significant term.

**Table 4:** TLBO results for individual response objectives.

Criteria	Design Variables			Predicted Results		Experimental Results		% Deviation	
	T_on_	T_off_	Current	MRR	SR	MRR	SR	MRR	SR
Maximization of MRR	95	9	2	1.114	3.80	1.119	3.69	4.55	2.98
Minimization of SR	35	27	2	0.599	2.81	0.608	2.85	1.54	1.75

**Table 5:** Non-dominated unique solutions obtained from TLBO.

Sr. No.	T_on_ (µs)	T_off_ (µs)	Current (A)	MRR (mm^3^/s)	SR (µm)
1	35	27	2	0.599	2.80
2	95	9	2	1.114	3.80
3	93	9	2	1.098	3.78
4	90	9	2	1.075	3.74
5	76	11	2	0.948	3.60
6	78	9	2	0.990	3.64
7	87	9	2	1.053	3.71
8	42	27	2	0.623	2.85
9	39	27	2	0.612	2.83
10	64	26	2	0.730	3.16
11	84	9	2	1.031	3.68
12	71	22	2	0.798	3.36
13	81	9	2	1.010	3.66
14	74	13	2	0.909	3.56
15	75	12	2	0.929	3.58
16	77	10	2	0.969	3.62
17	49	27	2	0.651	2.91
18	58	27	2	0.693	3.03
19	46	27	2	0.639	2.88
20	56	27	2	0.683	3.00
21	71	16	2	0.855	3.48
22	67	18	2	0.810	3.40
23	85	9	2	1.038	3.69
24	74	15	2	0.885	3.52
25	45	27	2	0.635	2.87
26	50	26	2	0.661	2.97
27	69	27	2	0.753	3.23
28	73	18	2	0.846	3.46
29	67	27	2	0.742	3.19
30	75	17	2	0.869	3.51
31	72	13	2	0.897	3.54
32	74	12	2	0.922	3.57
33	80	9	2	1.003	3.65
34	94	9	2	1.106	3.79
35	71	27	2	0.765	3.27
36	54	27	2	0.674	2.97
37	63	27	2	0.719	3.11
38	67	17	2	0.821	3.42
39	60	27	2	0.703	3.06
40	66	27	2	0.736	3.17
41	59	27	2	0.698	3.05
42	74	26	2	0.789	3.35
43	70	17	2	0.838	3.45
44	55	27	2	0.679	2.99
45	71	15	2	0.866	3.50
46	71	20	2	0.815	3.41
47	72	27	2	0.771	3.29
48	89	9	2	1.068	3.73

## Nomenclature

ANOVA	Analysis of variance
BBD	Box–Behnken design
DF	Degree of freedom
DOE	Design of Experiments
EDM	Electrical Discharge Machining
IEG	Inter-electrode gap
MOTLBO	Multi-objective teaching–learning based optimization
MRR	Material removal rate (mm^3^/s)
NDEDM	Near dry electrical discharge machining
NDWEDM	Near dry wire electrical discharge machining
RSM	Response surface methodology
SEM	Scanning electron microscope
SMA	Shape memory alloy
SMAs	Shape memory alloys
SME	Shape memory effect
SR	Surface roughness (µm)
TEM	Transmission electron microscope
TLBO	Teaching–Learning based optimization
T_on_	Pulse on time (µs)
T_off_	Pulse off time (µs)
t	Time in seconds
WEDM	Wire electric discharge machine
ρ	Density in g/cm^3^
